# A rare case of COVID-19 pneumonia with severe hyperlipoproteinemia

**DOI:** 10.1093/omcr/omac030

**Published:** 2022-04-19

**Authors:** Masafumi Shimoda, Yoshiaki Tanaka, Kozo Morimoto, Hiroyuki Kokutou, Taro Abe, Miyuri Suga, Takashi Yoshiyama, Kozo Yoshimori, Ken Ohta

**Affiliations:** Respiratory Disease Center, Fukujuji Hospital, Japan Anti-tuberculosis Association, Kiyose City, Tokyo, Japan; Respiratory Disease Center, Fukujuji Hospital, Japan Anti-tuberculosis Association, Kiyose City, Tokyo, Japan; Respiratory Disease Center, Fukujuji Hospital, Japan Anti-tuberculosis Association, Kiyose City, Tokyo, Japan; Respiratory Disease Center, Fukujuji Hospital, Japan Anti-tuberculosis Association, Kiyose City, Tokyo, Japan; Respiratory Disease Center, Fukujuji Hospital, Japan Anti-tuberculosis Association, Kiyose City, Tokyo, Japan; Respiratory Disease Center, Fukujuji Hospital, Japan Anti-tuberculosis Association, Kiyose City, Tokyo, Japan; Respiratory Disease Center, Fukujuji Hospital, Japan Anti-tuberculosis Association, Kiyose City, Tokyo, Japan; Respiratory Disease Center, Fukujuji Hospital, Japan Anti-tuberculosis Association, Kiyose City, Tokyo, Japan; Respiratory Disease Center, Fukujuji Hospital, Japan Anti-tuberculosis Association, Kiyose City, Tokyo, Japan

## Abstract

A 55-year-old woman was admitted to our hospital for coronavirus disease 2019 (COVID-19) pneumonia. Her symptoms improved upon treatment with steroids, remdesivir and heparin. After discharge, she consumed excessive alcohol because of taste disorder due to COVID-19; she also had chylomicronemia with a triglyceride (TG) level of 8750 mg/dl. Chylomicrons and very-low-density lipoprotein were detected by electrophoresis, and she was diagnosed with severe hyperlipoproteinemia, suspected to be Type V hyperlipoproteinemia. She did not have any symptoms of pancreatitis, and her TG levels decreased with fat intake restriction and sobriety. This is a rare case of COVID-19 with hyperlipoproteinemia, and the causes of hyperlipoproteinemia might be associated with COVID-19 complications, steroids and/or lifestyle changes during the pandemic; therefore, changes in TGs should be observed carefully after the resolution of COVID-19.

## INTRODUCTION

Coronavirus disease 2019 (COVID-19) caused by novel severe acute respiratory syndrome coronavirus 2 (SARS-CoV-2) has caused a worldwide pandemic, including pneumonia with multiorgan disease and various complications [1]. Many complications due to COVID-19 have been reported, even after discharge [2]. However, hyperlipoproteinemia combined with COVID-19 has not yet been reported; moreover, steroid therapy for COVID-19 can induce hyperlipidemia [1]. To the best of our knowledge, there is no report of severe hypertriglyceridemia >5000 mg/dl triglyceride (TG) appearing after COVID-19 [3]. We herein report a rare case of COVID-19 pneumonia combined with severe hypertriglyceridemia based on a TG level >8000 mg/dl, suspected to be Type V hyperlipoproteinemia.

## CASE PRESENTATION

**Figure 1:**
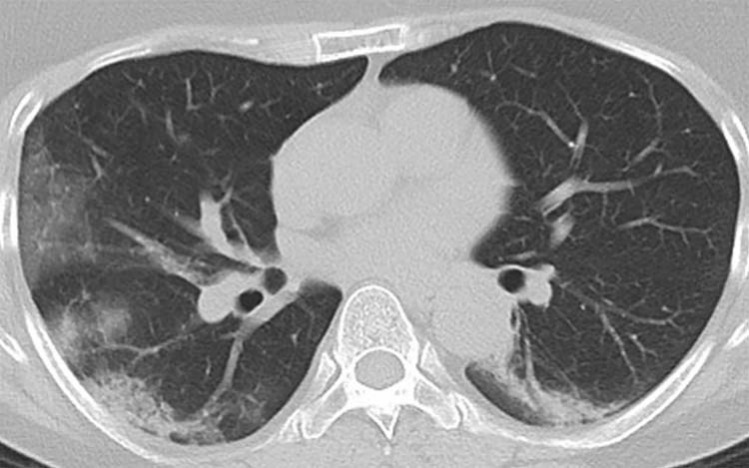
Chest CT scans showed multifocal bilateral peripheral ground-glass opacities.

A 55-year-old woman was admitted to our hospital for fever, cough, headache and taste disorder 6 days prior. She had a medical history of hypertriglyceridemia. She had increased her alcohol consumption during the COVID-19 pandemic since 2019, and treatment with 0.2 mg of pemafibrate was started because her TG levels had increased to ~700 mg/dl at 1 month before admission. However, lipid-lowering therapy was stopped by a local doctor due to the appearance of symptoms. There was no remarkable family history. Her vital signs on admission revealed a high-grade fever of 38.6°C and hypoxemia requiring oxygen at 2 l/min via nasal cannula. Physical examination did not show abnormal findings. The laboratory findings were as follows: white blood cell count of 3390 cells/μl; C-reactive protein level of 9.23 mg/dl; aspartate aminotransferase (AST) level of 78 IU/l; alanine aminotransferase (ALT) level of 50 IU/l and hemoglobin A1c (HbA1c) level of 5.9%. A positive result was obtained for SARS-CoV-2 detection by polymerase chain reaction using posterior oropharyngeal saliva samples. Chest computed tomography (CT) scans showed multifocal bilateral peripheral ground-glass opacities (Fig. 1).

Accordingly, she was diagnosed with COVID-19 pneumonia and treated with 60 mg of methylprednisolone, remdesivir and heparin. Her symptoms and radiographic findings improved gradually; however, the improvement was slow, and her radiological abnormalities remained on Day 10 of hospitalization. Therefore, steroids were not stopped on Day 10 of hospitalization, and the dose of steroids was decreased. Taste disorder remained after discharge, and she consumed excessive alcohol and a larger amount of food. At 32 days after the appearance of her initial symptoms, the blood sample appeared lipemic on 5 mg of prednisolone, and laboratory findings showed the following: TG level of 8750 mg/dl, low-density lipoprotein cholesterol level of 192 mg/dl, AST level of 106 IU/l, ALT level of 134 IU/l, amylase level of 50 U/l, lipase level of 36 IU/l and HbA1c level of 6.3%. Her thyroid function was normal, and antinuclear antibody was negative. Electrophoresis of the lipoproteins showed that the proportion of alpha was 9.3% (normal range: 20.4–46.4%), that of preβ was 41.8% (normal range: 3.8–33.8%), that of β was 25.6% (normal range: 36.5–59.1%) and that of the origin was 23.3%, which showed the presence of chylomicrons and an increase in very-low-density lipoprotein (VLDL) levels. The apolipoprotein fraction indicated concentrations of apolipoprotein B of 186 mg/dl, C-II of 47.5 mg/dl, C-III of 55.1 mg/dl and E of 32.2 mg/dl, and lipoprotein lipase (LPL) levels decreased to 50 mg/dl. The phenotype of apolipoprotein E was E3/2. The patient was thus diagnosed with hypertriglyceridemia, suspected to be Type V hyperlipoproteinemia. She did not have any symptoms of pancreatitis, and her TG levels were decreased by restriction of fat intake and sobriety.

## DISCUSSION

We herein report a rare case of COVID-19 pneumonia with severe hypertriglyceridemia, suspected to be Type V hyperlipoproteinemia. Generally, Type V hyperlipoproteinemia, which is a rare form of severe hypertriglyceridemia characterized by increasing VLDL and chylomicronemia, is caused by a genetic disorder or environmental factors such as heavy drinking and autoimmunity [4]. Regardless, she did not have a remarkable familial history, and the cause of Type V hyperlipoproteinemia was unknown. Hypertriglyceridemia is a risk factor for the development of acute pancreatitis [5]; therefore, changes in TGs should be observed carefully in patients after resolution of COVID-19 pneumonia.

One of the reasons for hyperlipoproteinemia progression might be the interruption of lipid-lowering therapy. The prevalence of hyperlipidemia in patients with COVID-19 is 26%, and lipid parameters, including TG levels, often decrease in the presence of cytokine-mediated inflammation as a consequence of the acute phase response [6]. It is suggested that lipid-lowering therapy should not be stopped for patients with COVID-19 [6]. Nevertheless, considering the slow progression of hyperlipoproteinemia until admission, it was thought that steroid therapy, complications of COVID-19 and/or lifestyle changes due to the COVID-19 pandemic might be associated with hyperlipoproteinemia progression in this case.

TG levels are influenced by several medications, such as steroids [7], which are used for COVID-19 treatment [1]. A previous study demonstrated that TG levels were increased in the long term to 156 ± 54 mg/dl by prednisolone treatment [7]. Some patients have TG levels > 1000 mg/dl, which is caused by corticosteroids, although none have TG levels > 2200 mg/dl [8]. Therefore, it is suggested that steroid therapy alone cannot increase TG levels to >8000 mg/dl. Furthermore, for patients with COVID-19, corticosteroids reduce 28-day mortality and are usually applied in the short term [1]. Our patient stopped steroid therapy on Day 39 through a decrease in dose. Accordingly, the cause of the increased TG level in our patient might be not only a side effect of corticosteroids but also other environmental factors.

Her alcohol consumption and amount of food intake increased during the COVID-19 pandemic, with a 4-fold declaration of a state of emergency in Tokyo, Japan, starting in April 2020. In general, people worldwide have had to stay at home and practice social distancing during the pandemic, and lifestyle changes are related to body weight gain and alcohol consumption [9, 10]. Alcohol with plasma TG metabolism leads to hypercholesterolemia in a complex interplay with partially altered LPL activity, often with a genetically predisposed background [4]. Moreover, she consumed alcohol excessively and a larger amount of food because of taste disorder as a COVID-19 complication after discharge. Accordingly, it is important to understand the changes in the patient’s lifestyle due to the COVID-19 pandemic and after the resolution of COVID-19.

This is a rare case of COVID-19 with Type V hyperlipoproteinemia. COVID-19 can induce many complications in accordance with therapy and social changes during the pandemic. Therefore, changes in TGs should be observed carefully in patients after the resolution of COVID-19 pneumonia.
